# Performance evaluation of a bi-directional synchronous H6 inverter for AC/DC system interaction with SiC and Si-Based at different switching frequencies

**DOI:** 10.1371/journal.pone.0304595

**Published:** 2024-12-17

**Authors:** Meshari S. Alshammari, Alireza Namadmalan, Maeve Duffy

**Affiliations:** 1 Electrical Engineering Department, College of Engineering, Jouf University, Sakaka, Aljouf, Saudi Arabia; 2 Power Electronics Research Center, University of Galway, Galway, Ireland; University of Cagliari, ITALY

## Abstract

The bidirectional inverter connected to the grid is a crucial component of DC distribution systems, however its operation can have an impact on the systems’ overall efficiency. The usual load profile of such systems in residential buildings is quite dynamic, with multiple periods of light load, especially when compared to high-demand sectors. This study examines and contrasts the impact of SiC and Si power MOSFETs on the best configuration of a 5 kW bidirectional H6 inverter specifically designed for residential use applications. Analytical modeling based on PSIM simulation results is employed to predict losses in a transformer-less synchronous H6 topology. Results of a 5 kW system indicate that silicon carbide (SiC) has an efficiency of up to 98.3%, surpassing the 93.6% efficiency attained with silicon (Si). Furthermore, this study explores potential of further improving efficiency by increasing the operating frequency to 50 kHz. The increase in frequency also leads to a reduction in the size of passive components. The experimental findings of a 1 kW system corroborate the efficacy of the proposed bidirectional synchronous H6 inverter topology, by demonstrating roughly the same trend in terms of the level of improvement over the baseline performance. It has been established that the use of SiC MOSFETs is superior to the use of Si MOSFETs, and that the appropriate operating frequency for bidirectional synchronous and comparable applications is 20 kHz rather than 50 kHz.

## 1. Introduction

**Fig 1:**
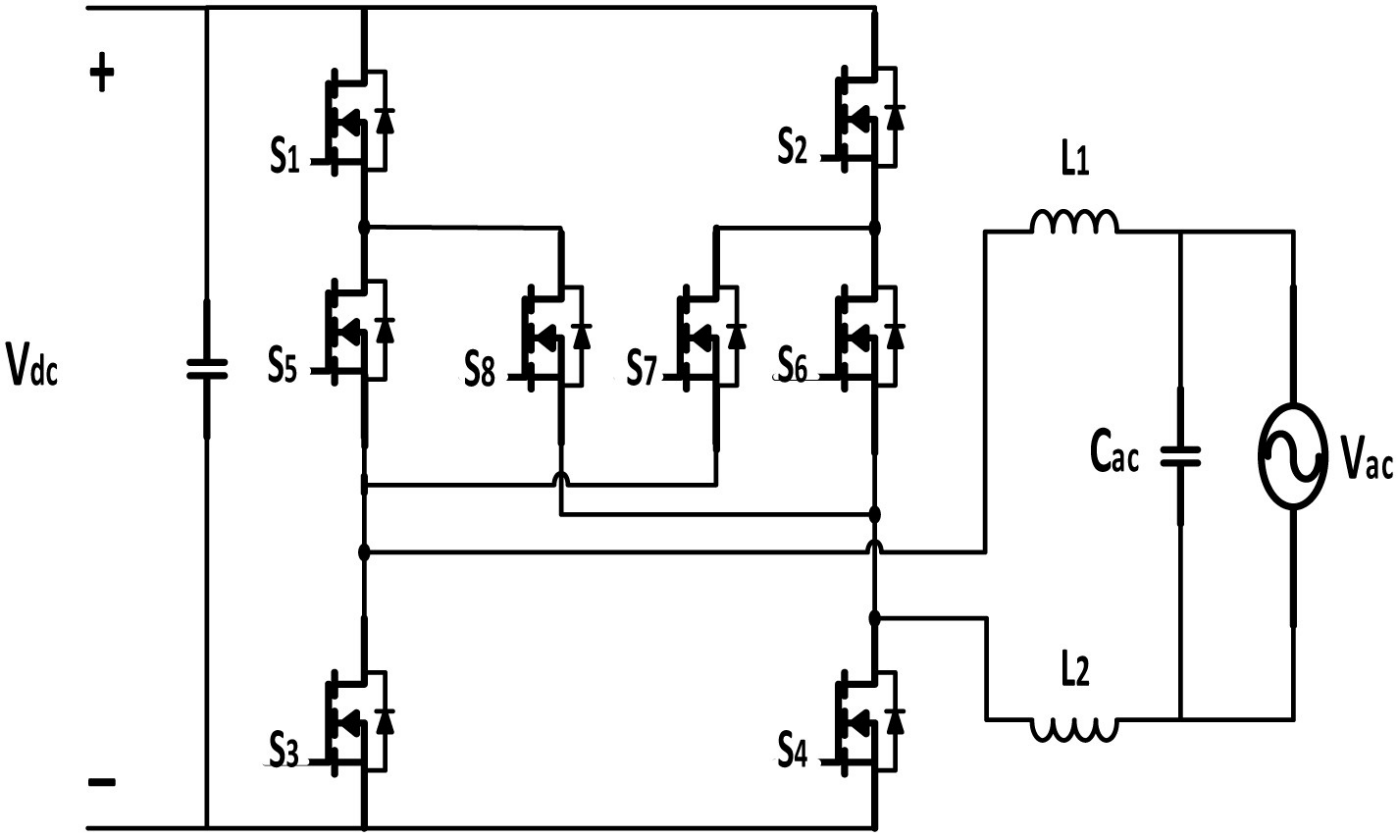
Synchronous bidirectional inverter topology.

Since DC distribution systems, particularly when combined with renewable energy sources, have the potential to be more efficient than AC systems, they might play a significant role in improving home energy efficiency. According to a recent study [1], the DC system demonstrates promising potential in lowering losses by up to 50% and achieving a 5% increase in grid energy savings when compared to its AC equivalent. Nevertheless, the requirement for power backup from the electrical grid necessitates the use of a bidirectional inverter to uphold the voltage of the DC bus while subjected to varying loads. Research findings suggest that the typical electricity consumption for residential dwellings is roughly 4200 kWh per annum, with the highest levels observed during the winter season, particularly in regions with a climate similar to that of Ireland [2]. A high-efficiency synchronous H6 bidirectional inverter (Fig 1) was thus proposed by the authors of [3] to reduce the negative impact on light-load efficiency. By utilizing SiC MOSFETs, they were able to reach an impressive efficiency of 98.3% at a load of 20%.

Silicon (Si) have been utilized in transformer-less topologies for applications with operating voltages below 1 kV, with the aim of attaining enhanced cost-effectiveness [4]. However, the losses in Si increase when the voltage and frequency are increased, whereas silicon carbide (SiC) has superior efficiency when compared to Si, mostly due to its reduced switching loss [5]. On the other hand, there are some challenges that associate from SiC’s fast dynamic response, such as voltage overshoot and the complicated interaction between the parasitic inductance and capacitance of the MOSFET body diode [6, 7]. Authors [8] have demonstrated the alleviation of these issues with the inclusion of a required dampening resistor for the SiC gate. The primary aim of this investigation is to evaluate the extent to which SiC improves performance relative to Si in a high-efficiency bidirectional grid inverter. The purpose is to enhance the decision-making process by enabling a comprehensive evaluation of the costs and benefits related to different bidirectional power flow applications. This will ultimately contribute to the assessment of the sustainability of integrating a DC distribution system with the utility grid.

Notably, SiCs are employed in different applications to fulfill a wide variety of power needs. Along with the ability to provide high voltages of up to 1,200 volts, SiC devices can also carry a substantial current [9]. Due to its inherent adaptability, this technology demonstrates suitability for a diverse range of applications, including grid-connected inverters and high-power three-phase grid converters [10, 11]. In comparison with SiC, the newly developed gallium nitride (GaN) devices have the potential to achieve efficiency levels higher than SiC devices [12]. GaN devices are typically rated for under 600-V and lower current than SiC, but are widely utilized to produce converters with power of more than 10 kW [13]. Despite this, SiC MOSFETs are chosen over GaN in this comparison owing to GaN’s significantly high cost and limited low operating frequency for high voltage devices (< 50 kHz), making it unsuitable for residential bidirectional inverter applications.

Given the high performance of SiC, it is expected that a high operational frequency might contribute to improving the performance and reducing the size and cost of passive components in a bidirectional converter. Inverters with higher operating frequency are used in variable-frequency AC sources. Energy storage systems (ESS) and electric vehicles (EVs) are among the applications for which higher operating frequencies are required [14–17]. Additionally, transmission of high-frequency AC within a microgrid is crucial for integrating distributed energy sources into a smart grid [18–20]. The power ratings of these inverters might vary from a few watts to a few kilowatts or more. The need for power electronics that are both more compact and more efficient in their operation has led the need for large improvements in switching frequencies.

By increasing the switching frequency, the feasible transient performance increases considerably. It also potentially allows for a reduction in the size of passive components whilst simultaneously increasing the integration of those components. However, this benefit is only accessible with passive components and circuit designs that are capable of operating effectively at the required frequencies. Therefore, this paper (as an extension of [21]) presents a framework for exploring optimization of the operating frequency of the proposed synchronous bidirectional inverter to enhance its performance in a DC distribution system. As a benchmark, the performance is compared with a Si implementation of the same converter, where Si is most widely used in bidirectional inverters for building applications.

The remainder of the paper is organized as follows: Section II presents a design procedure for the proposed bidirectional synchronous H6 as a function of frequency. This comprises of the analysis and design concerns for the synchronous H6, including the selection of components and their related power losses of SiC and Si technology. Simulation results are presented in Section III for a range of operating frequencies using SiC and Si technology, demonstrating the performance of the synchronous H6 inverter as well as its associated power loss. Experiments to verify the model predictions and to demonstrate the synchronous H6 system performance are discussed in detail in Section IV. Section V provides concluding remarks on the proposed work.

## 2. SiC vs. Si synchronous H6 analysis

**Fig 2:**
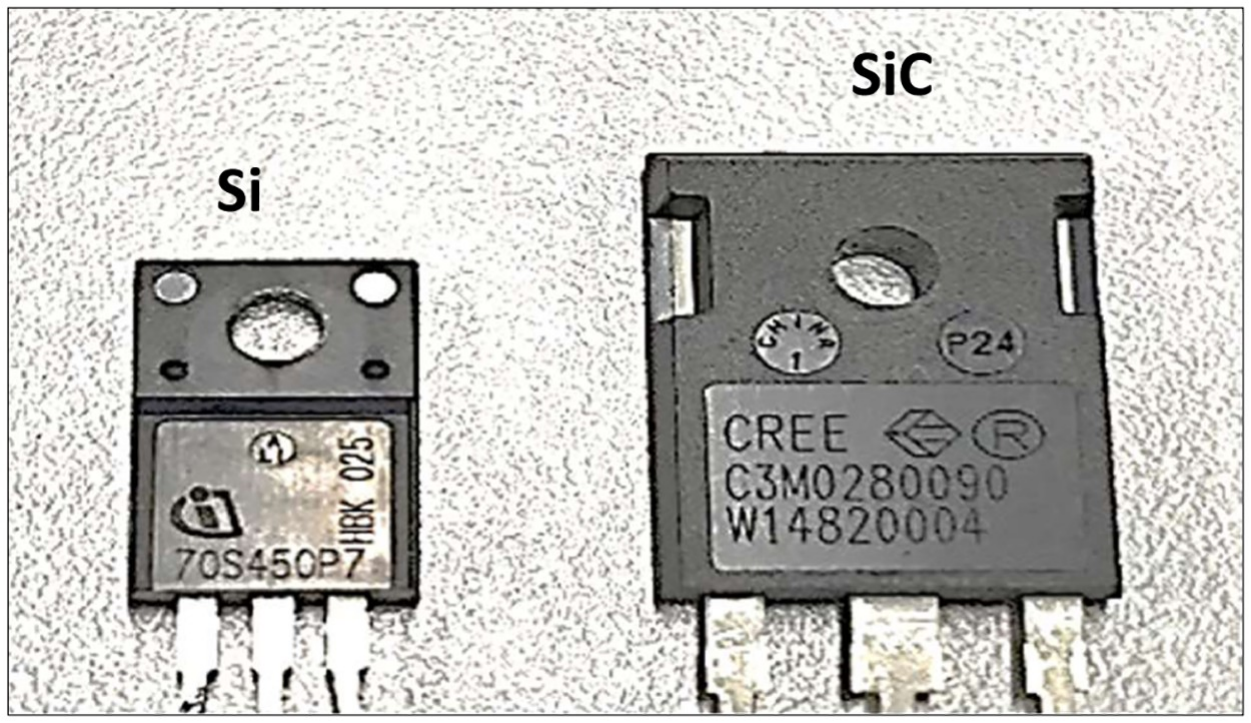
Illustration of Si and SiC MOSFETs used in the proposed bidirectional synchronous H6 inverter.

In this section, a simulation investigation is undertaken on the synchronous H6 architecture [3] using PSIM software. This study employs a set of extensive mathematical equations to evaluate the losses experienced by two distinct types of switching devices, namely (SiC) and (Si) as shown in Fig 2. The primary aim of this study is to enhance the overall operational efficiency of the bidirectional inverter. This study further investigates the potential for improving the efficiency of the synchronous H6 by increasing its operational frequency.

Since the development of the SiC MOSFET, it is often possible to directly replace Si with SiC MOSFETs in grid-integrated power converters. The SiC technology reduces switching losses, has low channel resistance and can work over a wider range of temperatures [22]. Due to its higher charge mobility and higher oxide dependability, it has a reduced ON resistance compared to conventional Si transistors. It is possible to achieve a lower RDS(on) in the high voltage layer or drift zone by using SiC vertical power MOSFETs because of their higher electric field, Emax, and lower doping level, allowing for a smaller thickness in the high voltage layer or drift region [23].

**Fig 3:**
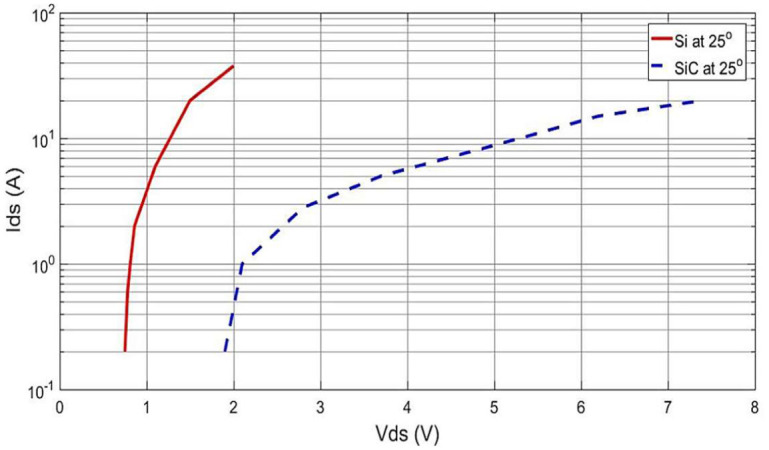
Measured forward voltage drops of Si and SiC MOSFET intrinsic diodes, as provided by the manufacturers’ data.

In relation to the intrinsic body diode, it is worth remarking that SiC has a higher forward voltage than Si for the same current level which results in higher turn-on losses and conduction losses during rectifier mode for SiC [3]. This effect may be seen in SiC MOSFETs operating at 600 and 1200 volts, although it is not generally significant [24–27]. According to Fig 3, the forward voltage of the chosen SiC body diode is five times greater than that of the Si device when both devices are working at their maximum capacities (i.e. 10 A). As a result, it should not come as a surprise that the diode conduction loss of SiC could be greater than that of Si which would result in a decrease in efficiency. On the other hand, the power loss is significantly impacted by the fact that Si has higher reverse recovery charge (Qrr) in comparison to SiC; e.g. Qrr of Si in this work is 700 nC versus 54 nC for SiC. In light of this, it is believed that this benefit will dominate over the issue of turn-on losses. This is certainly the case in an application such as a bidirectional inverter in buildings where the load typically tends to be low most of the time and losses are dominated by switching.

### Selected semiconductor devices

In order to compare the performance of the synchronous H6 at different operational frequencies, a DC operating voltage of 380 Vdc is utilized and the grid voltage and frequency of 220 V and 50 Hz respectively, are assumed for the AC side [21]. Listed in Table 1 are the semiconductors and passive components that were selected as the best suited for the specified working conditions, which include a maximum power rating of 5 kW and four different switching frequencies (Fsw) of 10 kHz, 20 kHz, 30 kHz and 50 kHz, respectively. This includes the SiC MOSFET model C3M0280090D manufactured by Cree, and as a best in class alternative, Infineon’s Si MOSFET IPA70R450P7S which combines the benefits of fast-switching and cost-effectiveness. In addition, it is equipped with an ESD protection diode across the gate-source that can decrease the turn-off time and therefore improve switching losses.

**Table 1:** Component choices of the synchronous h6 for frequencies from 20 kHz—50 kHz.

Components	Model No.	Rated Voltage (V)	Rated Current (A)
SiC MOSFET	*C3M0120090D*	*900*	*23*
Si MOSFET	*IXFH 24N80P*	*800*	*24*
Inductor– 10k Hz	*990 μ H—MPP 0055102A2*	-	-
Inductor—20k Hz	*470μ H—MPP 0055617A2*	-	-
Inductor—30k Hz	*333 μ H—MPP 0055868A2*	-	-
Inductor—50k Hz	*200μ H—MPP C055110A2*	-	-
Capacitor	*4*.*7 μF*	-	-
Capacitor type	*Film C4AF*	*250*	*16*.*6*

### Inductor design

With increasing frequency, it is possible to decrease the filter inductance by the same factor according to:

$$L=\frac{V_{dc}}{4Fsw\Delta I_{out}}$$ (1)

where *L* is the required inductance to minimize noise caused by the switching frequency *Fsw* for a synchronous H6 in which the maximum voltage and current under inversion and rectification modes were considered.

To determine the core size (volume) that is large enough to accommodate the required energy stored the following equation is applied to allocate the core size:

$$E>L\;I^{2}$$ (2)

where *E* the required stored energy, *L* is inductance required with DC bias (mH) and *I* is maximum current (A).

Using the energy calculated with Eq (2), a suitable core size is chosen according to inductor design tool provided by [28]. The next step is to select the permeability of the chosen core material. The selection criteria is based on choosing the smallest core size for application with a given DC bias, and low core loss when operated at a low-level of AC current relative to DC current. Then, the required number of turns can be obtained from Eq (3).


$$N=\sqrt{\frac{L\;10^{3}}{A_{l}}}$$ (3)

In the given context, N represents the quantity of turns, whereas *A*_*l*_ indicates the inductor factor associated with the chosen core. The determination of the maximum magnetic field intensity is performed in order to prevent core saturation, as outlined by Eq (4).

$$H=\frac{NI}{I_{e}}$$ (4)

where *I*_*e*_ is the core magnetic path length, and the result determines an initial permeability from the curve fitting of permeability vs. DC bias which is provided in the datasheet of the selected core. Iteration can be performed until a satisfactory number of turns is obtained in which the optimal inductor value can be adjusted to handle the performance.

**Fig 4:**
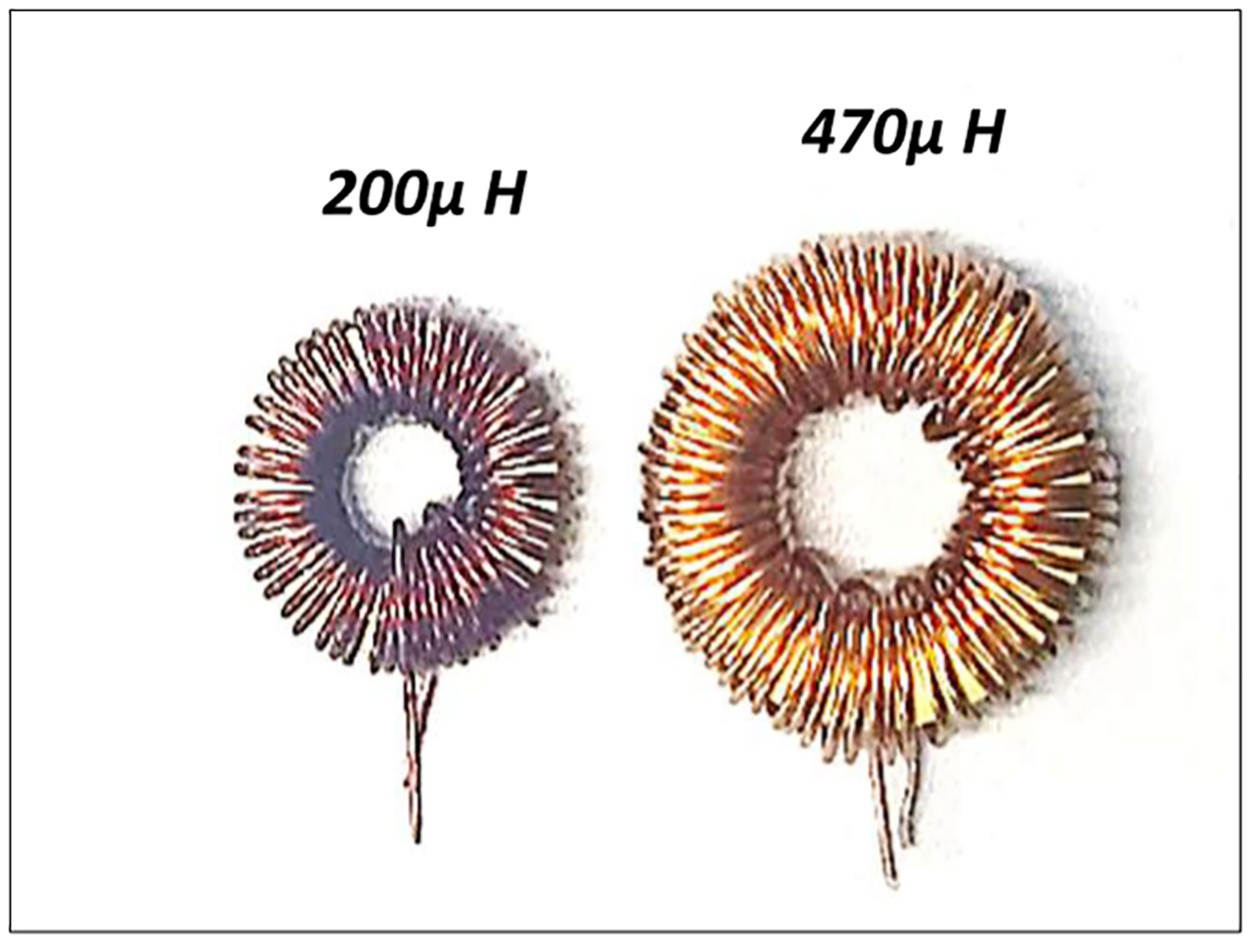
Illustration of utilised inductors for operation at 20 kHz and 50 kHz.

Then, the inductor equivalent series resistance can be calculated as follows

$$L_{resi}=N\;R_{wire}$$ (5)

where *R*_*wire*_ is the specified resistance of the inductor AWG wire per turn, which is selected based on its maximum current handling capacity. The total wire length is based on the core dimensions as illustrated in Fig 4. It is important to note that for final production and commercialization at large scale the Litz wire should selected.

### Loss calculation

Standard equations were applied for calculating the MOSFET and inductor losses based on the circuit operating waveforms simulated in PSIM, similar to the method described in [3, 21]. In this case, of course, loss parameters were identified from the Si datasheets when considering Si versus SiC losses, and losses in the inductors accounted for the new designs produced for each operating frequency.

Conduction loss of the SiC MOSFETs (*Pcm*) and MOSFET body-diodes, (*Pc*_*anti*_*d*) are given as:

$$Pcm=R_{dson\_s}\;I_{rms\_s}^{2}$$ (6)

$$Pc_{anti}d=V_{fsm}\;I_{av\_md}+\;R_{dson\_d}\;I_{rms\_md}^{2}$$ (7)

the parameter R_*dson_x*_ represents the on-resistance of the specified component, while I_*rmx*_*_x* represents its root mean square (rms) current. V_*fsx*_ corresponds to the forward voltage drop of the given diode component, and I_*av_x*_ defines its average current. The switching losses of SiC and Si MOSFETs, abbreviated as P_*swonm*_ and P_*swoffm*_, are provided.

$$Psw_{on}m=\left(V_{pkm\_on}\;I_{pkm\_on}\;\frac{Tr}{2}+Q_{rr}\;V_{pkm\_on}\right)\;F_{sw}$$ (8)

$$Psw_{off}m=\left(V_{pkm\_off}\;I_{pkm\_off}\;\frac{Tf}{2}\right)\;F_{sw}$$

where *Tr* & *Tf* represent the rise and fall times of the MOSFETs during the switching operation. *Q*_*rr*_ symbolizes the reverse recovery charge of the specified diode, while *V*_*pkm_x*_ and *I*_*pkm_x*_ are the forward voltage and current of the diode. Besides, *F*_*sw*_ stands for the carrier switching frequency. In Eq (9), the MOSFET gate charge losses are shown in terms of the electric charge (Q) and capacitance (C) of the high and low sides, which are given in the MOSFET datasheet.


$$Pg=\;((Q_{g-h/l}\;)\;V_{gs}\;F_{sw})+((C_{g-h/l}\;)\;V_{gs}^{2}\;F_{sw})$$ (9)

The calculations for copper and core losses (*Pcopper*, *Pcore*) in the filter inductors were performed in accordance with the specified approach.

$$Pcopper=I_{rms}^{2}\;R_{wire}$$ (10)

$$\text{Pcore}=\frac{\text{PL}\;\text{Ae}\;\text{Le}}{1000}$$ (11)

$$\text{PL}=31.32\;{\Delta \beta }^{1.585}\;{\text{F}}^{1.37}$$ (12)

where *Ae* represents the core’s cross-sectional area, *Le* represents its path length, and *PL* corresponds to the core loss density as a function of AC flux *Δ*β. F indicates the frequency of operation (k Hz), while k and c are material constants. The loss in equivalent series resistance (*ERS*) of the capacitor is expressed as

$$Pca=I_{rms}^{2}\;ERS$$ (13)

The choice of the DC link and AC capacitor were calculated according to:

$$C_{dc}\geq \;\frac{I_{load}}{8\pi \;V_{ripple}}$$ (14)

$$C_{ac}\geq \frac{I_{load}}{2\;F_{sw}\;V_{ripple}}$$ (15)

where, *I*_*load*_ is the current load, *V*_*ripple*_ is the required voltage ripple.

During each half cycle of the mains, the PSIM simulation was used to measure representative values of the peak, average, and rms voltages and currents for each semiconductor component at four different times [21]. These values were subsequently employed in Eqs (6)–(13) to determine the breakdown of circuit losses over different power levels.

## 3. Simulation results

### Comparison of SiC and Si performance

**Fig 5:**
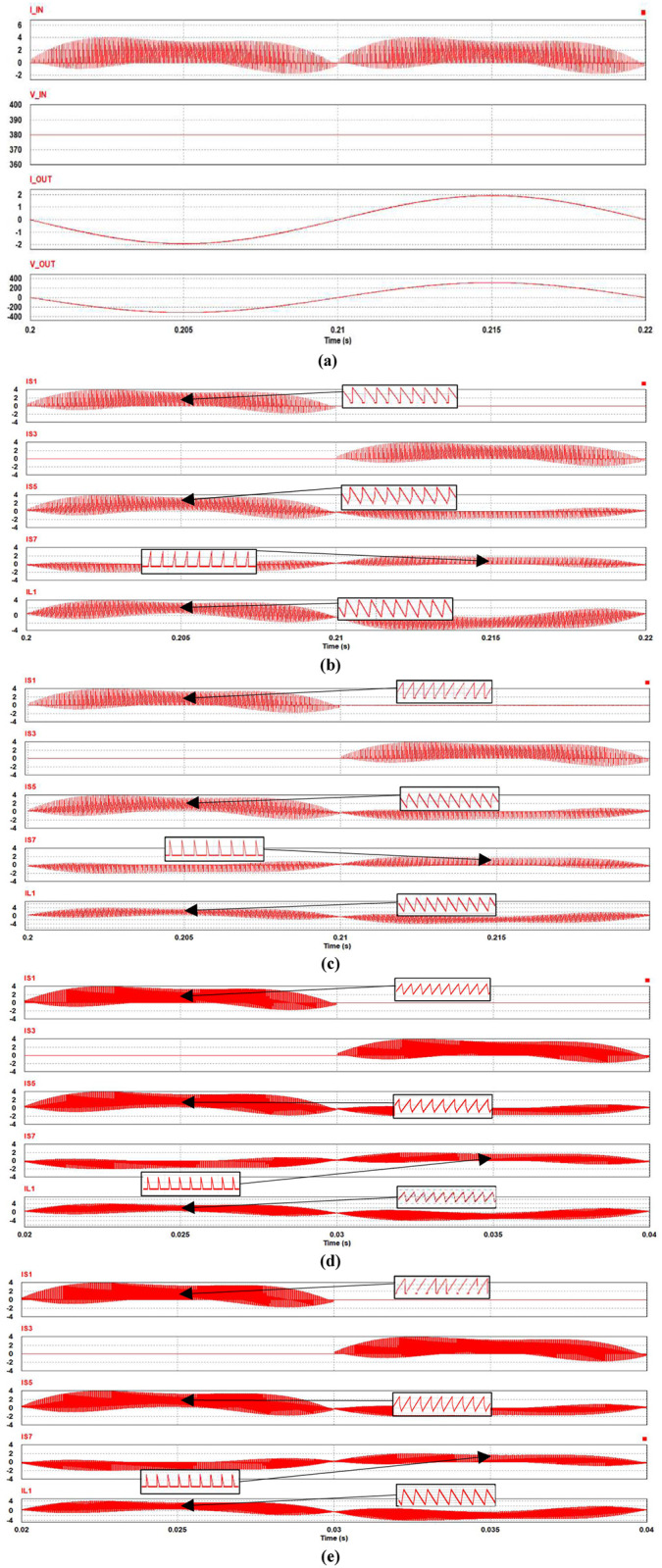
Performance of synchronous H6 operated in inverter mode at 20 k Hz: (a) Input voltage and current, (b) SiC switching and inductor current waveforms, (c) Si switching and inductor current waveforms, and at 50 kHz (d) SiC switching current waveform and (e) Si switching current waveforms.

The performance of the synchronous H6 topology with the implementation of SiC vs. Si MOSFET operated at two frequencies 20 kHz and 50 kHz are illustrated in this section. Loss calculations in previous section II are based on the operating current and voltage waveforms for all switches, which are shown for inversion mode at 20 kHz and over a complete cycle of the mains in Fig 5. Also, the inductor current waveforms are shown. It is important to note that the current and voltage waveforms of both SiC and Si are nearly the same, with no significant difference observed. Similarly, at a high frequency of 50 kHz, the current and voltage waveforms exhibit a similar pattern to those at 20 kHz.

**Fig 6:**
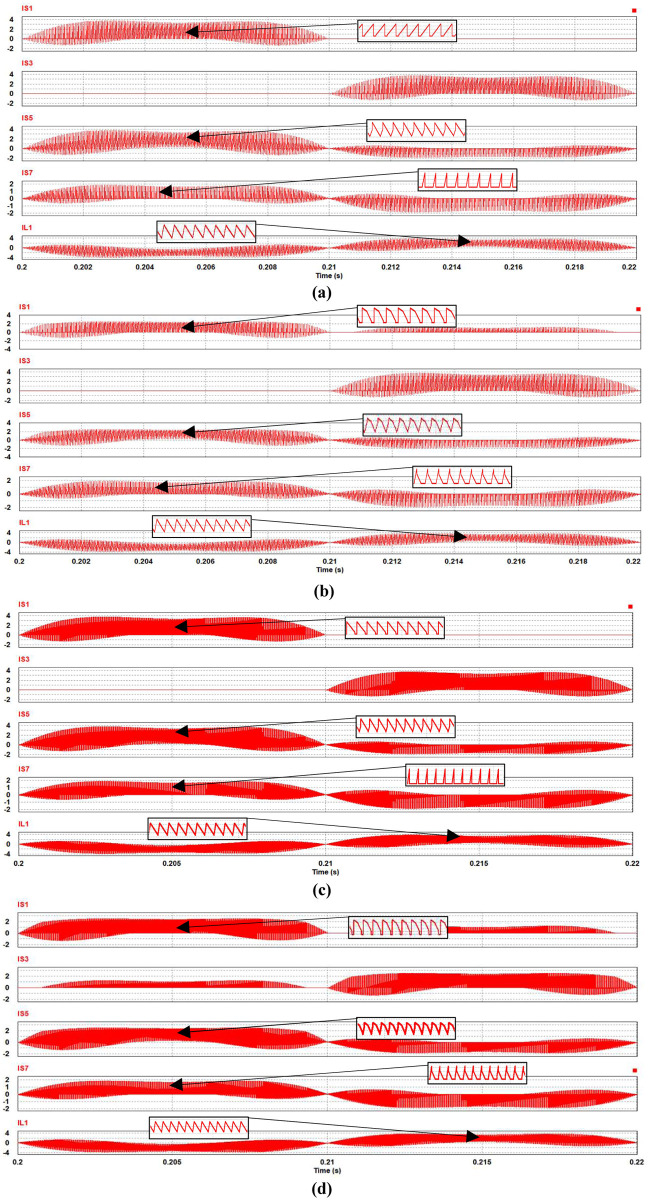
Performance of synchronous H6 operated in rectifier mode at 20 k Hz: (a) SiC switching and inductor current waveforms, (b) Si switching and inductor current waveforms, and at 50 kHz (c) SiC switching current waveform and (d) Si switching current waveforms.

Almost the same waveforms are found for the switches and inductors when operated in rectifier mode as shown in Fig 6. Operation at 50 kHz as shown in Figs 5c, 5d, 6c and 6d is found to have relatively similar waveforms, including for the inductor due to the design according to Eq (1) in section II which maintains its current ripple level. The switching waveforms of S1 and S2 are the mirror of S4 and S3 respectively. Similarly, the switching waveforms of S5 and S7 correspond to the waveforms of S6 and S8. To illustrate the current waveforms in each switch more clearly, zoomed in current waveforms are shown for each of the circuit switches at the peak current level in Figs 5 and 6.

It is worth mentioning that at this stage that the main difference between Si and SiC in terms of power loss is a higher on resistance for Si which contributes to additional conduction loss in both rectification and inverter modes. For SiC, there is more significant contribution of the MOSFET body-diode, which limits performance particularly in rectifier when the current path includes the body-diode to maintain the same PWM signalling scheme [3]. Furthermore, increasing the frequency not only reduces the loss in the inductor but also leads to a relatively higher loss in semiconductor switching, particularly during the off state. This phenomenon occurs due to the need to dissipate the energy stored within the body-diode of the MOSFET when it is being turned on. Therefore, as the frequency increases, there is a fundamental trade-off between losses in semiconductors, in particular MOSFETs, and losses in inductors. The aforementioned trade-off is expounded upon in the next section.

### Loss breakdown

A comparison of the loss breakdown for a 5 kW bidirectional synchronous H6 inverter with the implementation of Si (Rdson = 0.4 Ω) and SiC (Rdson = 0.12 Ω, see Table 1) respectively at four different frequencies: 10 kHz, 20 kHz, 30 kHz and 50 kHz, is presented in Fig 6. At all frequencies, the results show a significant contribution of Si conduction loss at higher power levels. In addition, the switching ON loss of Si is higher than SiC by up to 85% at 20% load where it is more dominant. It is clear that the significant copper losses in the inductors and the switching on loss contribute to the higher power loss of Si at full power when the operating frequency is 10 kHz as compared to the 20 kHz. This illustrates the trade-off between inductor and MOSFET losses mentioned above and applies for both Si and SiC designs. Nevertheless, switching on loss and MOSFET body-diode losses for Si account for most of the loss when operating above 30 kHz. However, the switching on loss for SiC, in comparison to Si, is the primary contributor to power loss when increasing the frequency up to 50 kHz.

Higher frequency also results in miniaturization of the inductor with up to 65% associated inductor loss reduction at full load. Therefore, while SiC retains a higher performance over Si, the overall improvement in efficiency achieved by increasing the frequency is limited at full load, and efficiency is actually reduced at light-load.

**Fig 7:**
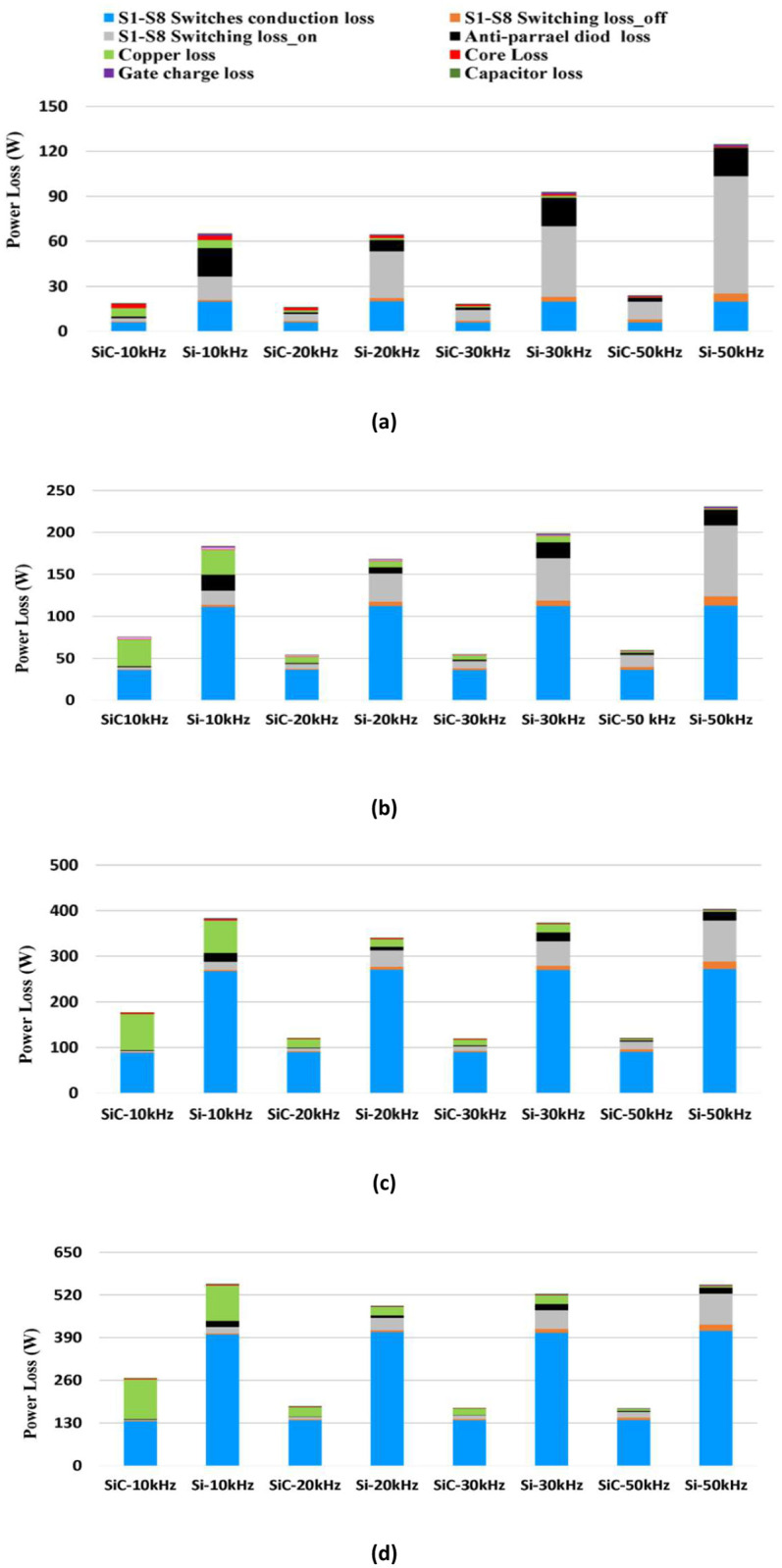
Comparison of power loss breakdown between SiC vs. Si MOSFETs in inversion mode operated at 10k, 20k, 30k and 50 kHz at; (a) 1 kW, (b) 2.5 kW, (c) 4 kW and (d) 5 kW.

**Fig 8:**
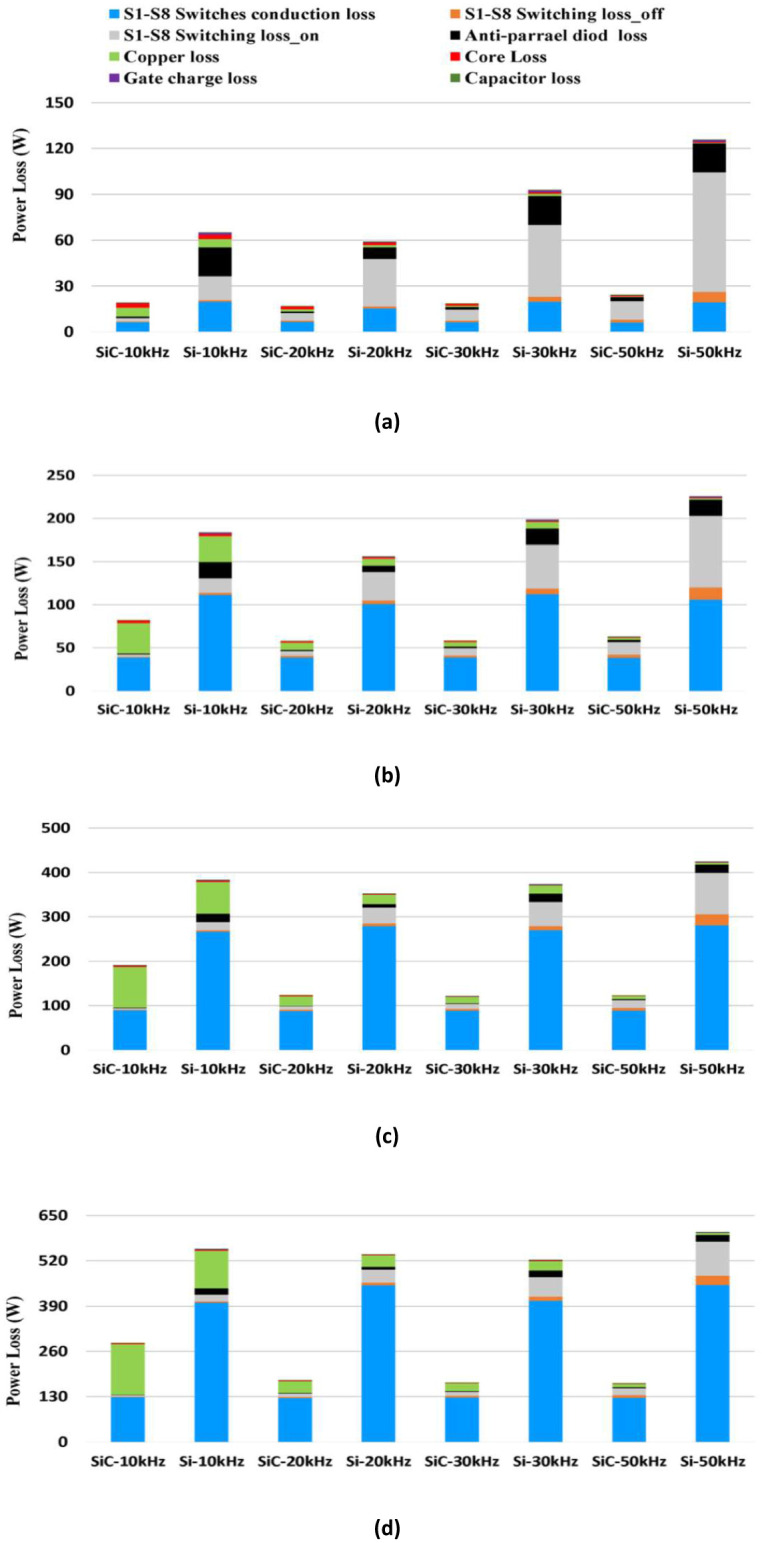
Comparison of power loss breakdown between SiC vs. Si MOSFETs in rectification mode operated at 10k, 20k, 30k and 50 kHz at; (a) 1 kW, (b) 2.5 kW, (c) 4 kW and (d) 5 kW.

Similarly, in rectifier mode, while the full load losses are relatively higher than inverter mode (see Fig 8), a significant improvement is found by employing SiC over Si, especially under light-load conditions as shown in Figs 7 and 8. It is found that the major drawback of Si is the reverse recovery effect which has an impact on the synchronous H6 topology when a freewheeling path is employed to maintain the inductor current during off state operation. In addition, the conduction loss is affected due to the characteristic of the reverse recovery charge which exists in the Si body-diode. Again, it is noticed that the performance of Si tends to decrease more significantly at high operational frequency than SiC due to the impact of high switching loss along with the body-diode effect enabled during the rectification mode.

### Efficiency comparison of Si vs. Sic

**Fig 9:**
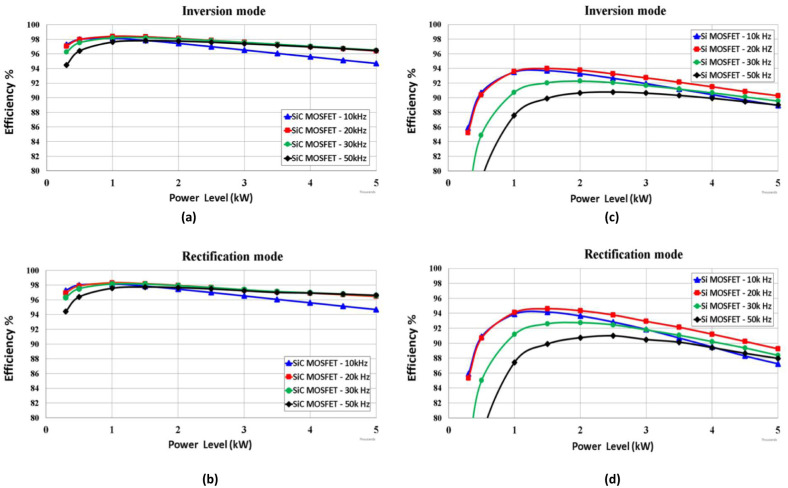
Efficiency comparison of SiC MOSFET at (a) inverter mode, (b) rectification mode and Si MOSFET at (c) inversion mode and (d) rectification mode operated at 10k, 20k, 30k & 50k Hz for 5 kW system.

**Fig 10:**
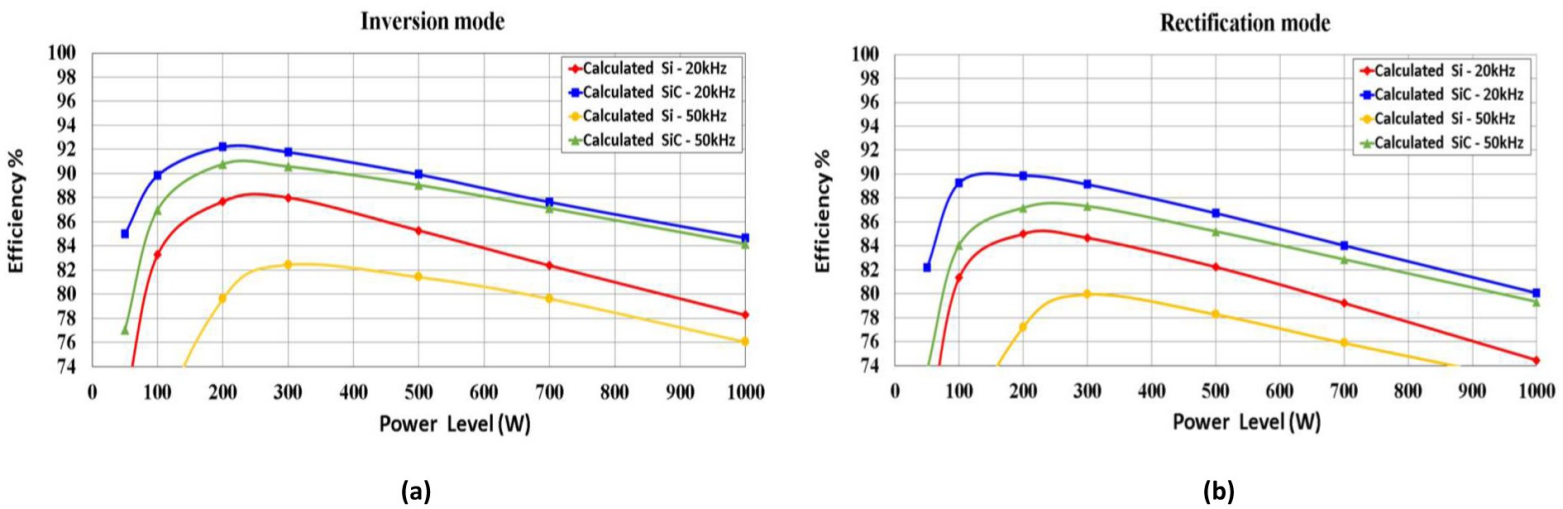
Efficiency comparison of SiC and Si MOSFET at (a) inverter mode, (b) rectifier mode operated at 20k Hz 50k Hz for a simulated 1 kW system.

A comparison of the efficiency of SiC vs. Si operating in the proposed synchronous H6 topology is presented in Fig 9 for the 5 kW system studied. In addition, Fig 10 illustrates the simulation results of a 1 kW system, which are verified in the following section by experimental results for the demonstrator prototype.

The improved efficiency provided by SiC compared to Si tends to be by far most significant when the operational power is reduced towards light-load. As shown in the results of loss breakdown above, this is largely due to MOSFET conduction loss under all power level, in addition to the switching ON when operated under light-load condition. As discussed in relation to losses, increasing frequency above 20 kHz does not provide improved performance.

To verify both the operation and design of the proposed 5 kW bidirectional synchronous H6 inverter, Fig 10 depicts a simulated prototype demonstration system with a power output of 1 kW. To ensure proper integration of the revised rated power findings for the prototype circuit, which will be explained upon in the following experimental section, all system components have been resized.

## 4. Experimental results

**Fig 11:**
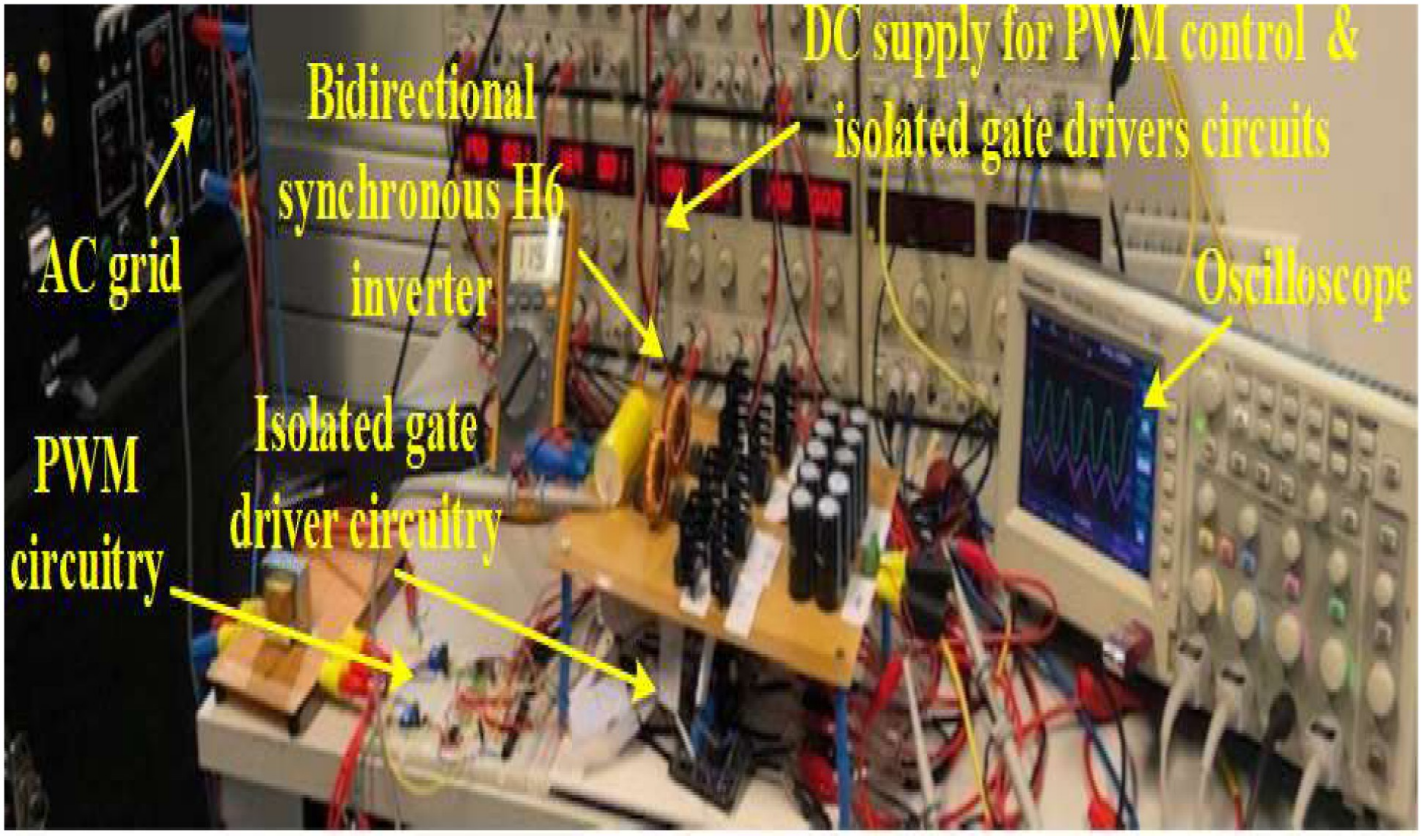
A hardware prototype test-bed setup of a 1 kW synchronous H6 inverter.

To validate the proposed 5 kW bidirectional synchronous H6 inverter, a scaled-down prototype demonstration system of 1 kW is built where all components were re-sized based on the new rated power. Table 2 shows the components chosen for a 1 kW system with 20 kHz and 50 kHz switching frequencies. Fig 11 illustrates the 1 kW resized bidirectional synchronous H6 inverter.

**Table 2:** Component choices of the synchronous H6 for frequencies from 20 kHz and 50 kHz.

Components	Model No.	Rated Voltage (V)	Rated Current (A)
SiC MOSFET	C3M0280090D	900	11.5
Si MOSFET	IPA70R450P7S	700	10
Inductor—20k Hz	470μ H (MPP core)	200	13
Inductor—50k Hz	200μ H (MPP core)	200	13
AC Capacitor AC	4.7 μF	250	12
DC Capacitor	3760 μF	220	13

**Fig 12:**
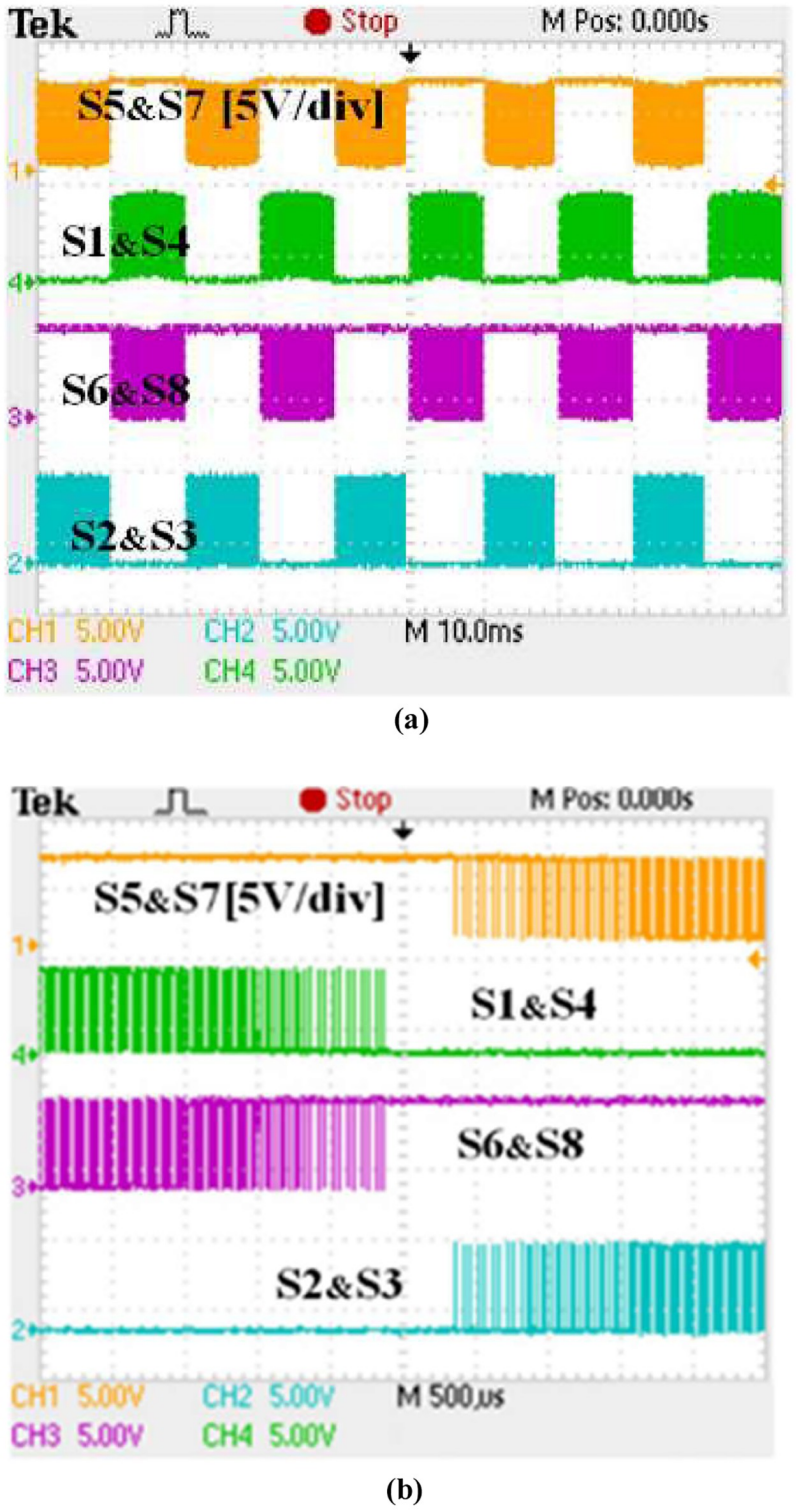
Synchronous H6 at inverter and rectifier modes (a) pulses of the PWM waveforms and (b) pluses of an expanded PWM waveforms.

Details of the implemented switching gate signals are shown in Fig 12. The switching pattern is the same regardless of whether the converter is in rectifier or inverter mode, demonstrating that the same PWM modulation signals are applied.

**Fig 13:**
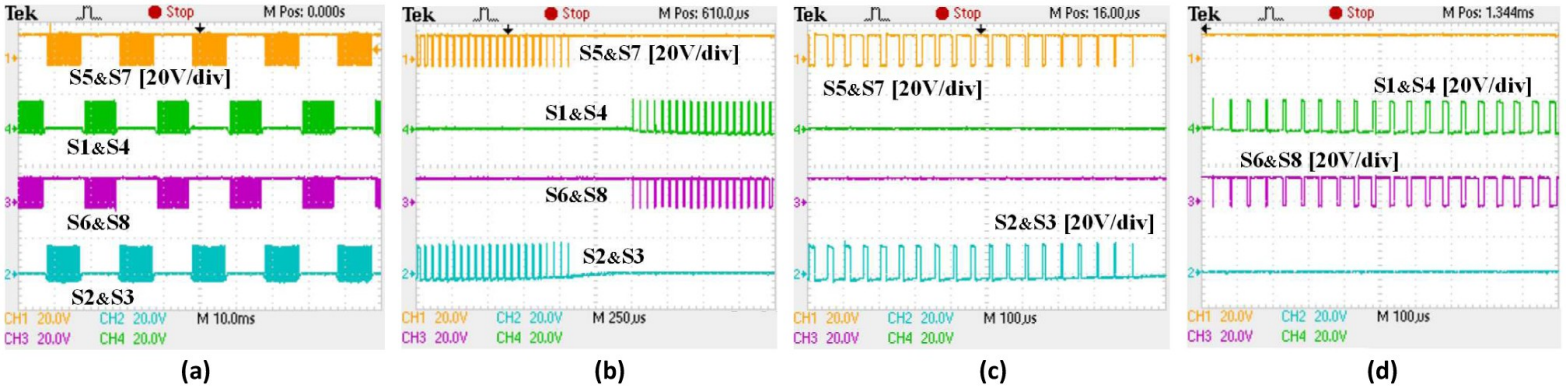
Switching pluses under 20kHz for used SiC and Si MOSFETs: (a) full switching pulses S1-S8, (b) expanded switching pluses S1-S8, (c) detailed switching pluses for S5&S7 and S2&S3 and (d) detailed switching pluses for S6&S8 and S1&S4.

**Fig 14:**
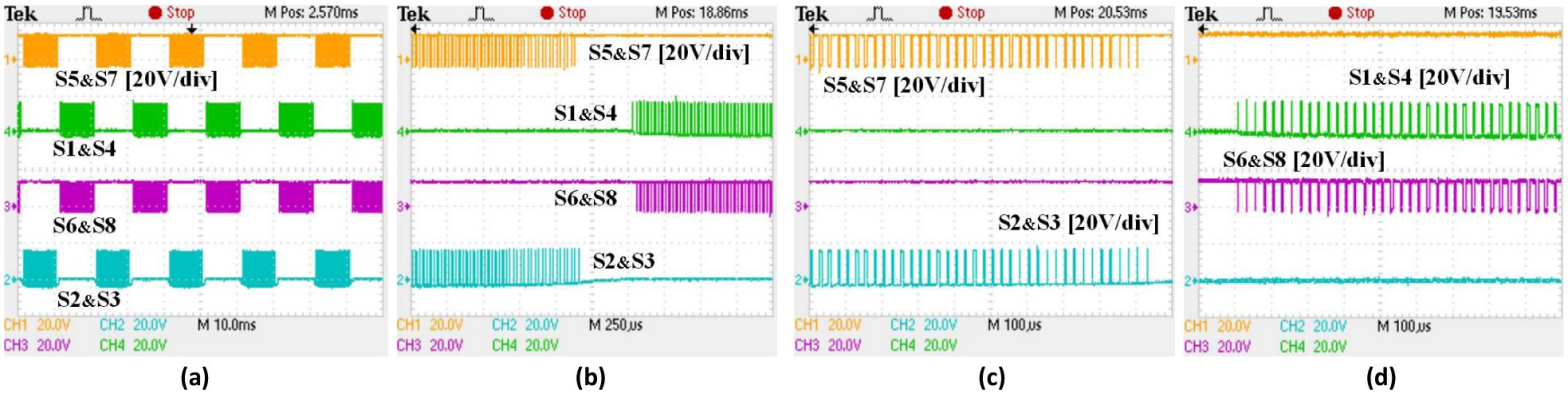
Switching pluses under 50kHz for used SiC and Si MOSFETs (a) full switching pulses S1-S8, (b) expanded switching pluses S1-S8, (c) detailed switching pluses for S5&S7and S2&S3 and (d) detailed switching pluses for S6&S8and S1&S4.

It is recommended that the C3M0280090D SiC MOSFET should be operated between (+15V and -4V) in order to drive it effectively, as compared to other switches such as the Si MOSFET, which don’t require a negative signal [29]. Despite this, Si MOSFETs are also capable of operating within the design limits of the SiC MOSFETs as it tolerates more negative voltage. As a result of this, a design circuitry including gate driver that provided the voltage range needed for the optimal operations has been integrated into the bidirectional inverter. As demonstrated in Figs 13 and 14, the PWM output signals for the employed power MOSFETs operated between -4V and 15V.

**Fig 15:**
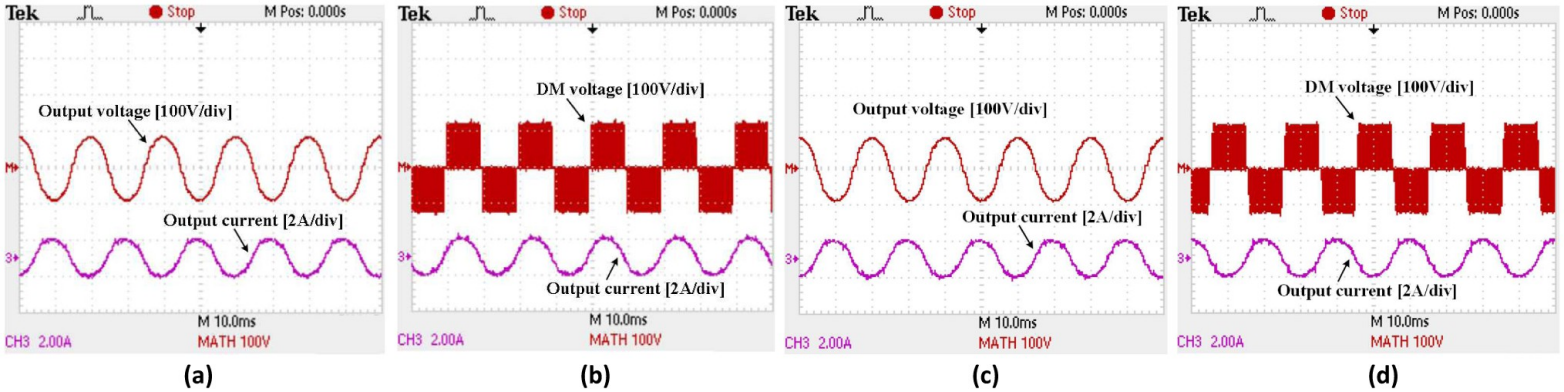
Measurement waveforms of synchronous H6 at inverter mode at 20kHz (a) output voltage and current, (b) differential mode (DM) voltage using SiC MOSFETs, (c) output voltage and current and (d) DM voltage using Si MOSFETs.

Fig 15a and 15c show the output voltage and AC current of a bidirectional synchronous H6 inverter that uses Si and SiC MOSFETs and operates in inverter mode at 20kHz and output a power of 50 W. These values demonstrate operation at the grid frequency of 50 Hz, whilst the modulation index is approximately 75%. The DC link voltage at the inverter’s output is around 120 V, and the inverter mode current and voltage waveforms are in phase for both Si and SiC MOSFETs. A limitation in performance at light-load is indicated by a low (THD) for both Si and SiC respectively. However, when the circuit is operated at a power level above 100 W, the THD of grid current decreases, bringing about a subsequent return to higher levels of efficiency.

The differential mode (DM) voltage and AC current of the synchronous H6 prototype operating in inverter mode utilizing Si and SiC MOSFETs is demonstrated in Fig 15b and 15d, respectively. The improvements of its DM voltage are demonstrated by the standard three-level voltages which are +1, 0 and -1, whilst the elimination of CM and leakage current typically seen in standard H6 topology (like the proposed synchronous topology) is verified by a constant DC with minimal AC ripple, while this also verifies the feature of eliminating the CM and leakage current encountered in H6 topologies, such as the proposed synchronous topology.

Further tests for the synchronous H6 prototype were carried out with an operating frequency of 50 kHz using both SiC and Si MOSFETs. In comparison with 20 kHz, SiC MOSFETs have 7.5% lower efficiency at 50 kHz with the same 50 W load. Nonetheless, this reduction in efficiency is still superior to the use of Si MOSFETs, in which the efficiency at 50 kHz is reduced by 21% when compared to 20 kHz. Particularly, this is due to the effect of the reverse recovery of Si MOSFETs due to the higher operating frequency. However, the effect of the reverse recovery of SiC is significantly smaller and when combined with lower switching loss, the use of SiC can substantially improve the efficiency of the synchronous H6. It is important to note however, that the conduction loss of the SiC body diode during freewheeling mode is higher than that of Si whilst operating at the same load due to the higher forward voltage drop. In spite of this, the SiC MOSFETs are still able to provide a lower power loss in comparison to the Si MOSFETs.

**Fig 16:**
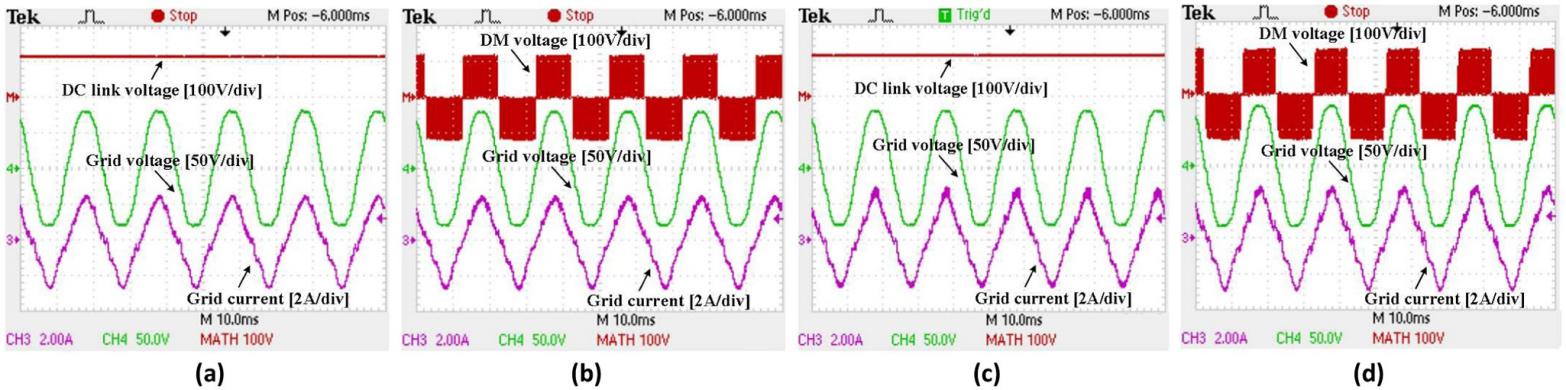
Switching pluses under 20kHz for used SiC and Si MOSFETs (a) full switching pulses S1-S8, (b) expanded switching pluses S1-S8, (c) detailed switching pluses for S5&S7and S2&S3 and (d) detailed switching pluses for S6&S8and S1&S4.

For rectifier mode, Fig 16a and 16c depict the input voltage, AC current and output DC link voltage of the bidirectional synchronous H6 using Si and SiC MOSFETs at 20 kHz at light-load in rectifier mode (50 W). These results demonstrate that the modulation index is approximately 66%. The DC link voltage is around 120 V at the rectifier’s output, and the power factor (PF) of the inverter mode waveforms for both Si and SiC MOSFETs is 1. A higher THD of grid current is observed at low load (50 W) in both Si and SiC compared to inverter mode. This is due to a low impedance path from the grid when the grid voltage has little THD. In addition, the THD of the grid voltage could vary depending on the grid supply. When the bidirectional synchronous H6 is operating at a power level more than 100 W, the THD of the grid current relatively decreases as before, leading to an efficiency increase.

The DM and DC link voltage as well as grid and voltage current of the synchronous H6 prototype operating in rectifier mode with Si and SiC MOSFETs are displayed in Fig 15b and 15d respectively. Similar to inverter mode, the normal three-level voltages of +1, 0 and -1 show upgrades to the DM voltage, with a constant DC although relatively higher AC ripple compared to the inverter mode. The elimination of CM and leakage current in H6 topologies, such as the proposed synchronous topology, is achieved.

Testing to compare SiC and Si MOSFETs in rectifier mode at an operating frequency of 50 kHz was also conducted. When running at 50 kHz with the same 50 W load, the performance of SiC MOSFETs drops by 8.9% compared to operating at 20 kHz. However, this is still an improvement over using Si. Again, the effect of the reverse recovery of Si increases with increasing frequency. As SiC has lower switching loss than Si, they thus consume less power overall. However, because of their high body diode forward voltage, SiC MOSFETs have a relatively higher conduction loss during freewheeling modes than Si MOSFETs when subjected to the same load. Despite this however, the SiC MOSFETs still deliver less power loss than that of the Si MOSFETs.

**Fig 17:**
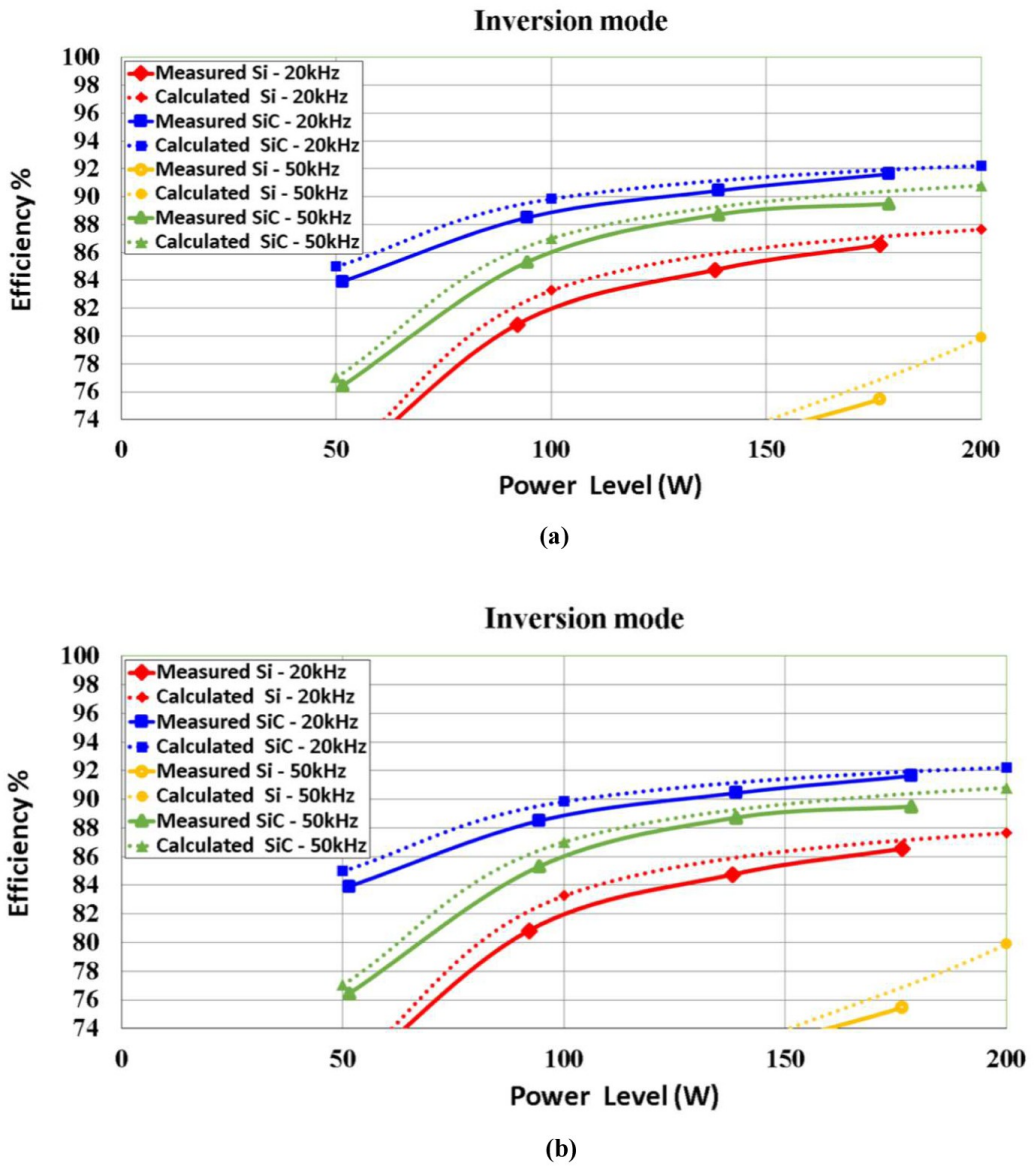
Efficiency curves of bidirectional synchronous H6 inverter at (a) inverter and (b) rectifier modes.

Last but not least, the inverter and rectifier efficiency curves for the bidirectional synchronous H6 inverter are shown in Fig 17. When operating at 20 kHz and 50 kHz, respectively, with 20% of the nominal load, the efficiency of the synchronous H6 in inverter mode utilizing SiC can be increased up to 14.8% and 26.6% when compared to the use of Si. The highest efficiency of a 1 kW synchronous H6 was calculated to be 92.7% in the inversion mode and 89.8% in the rectification mode. Although the claimed efficiency is higher than the observed efficiency at full load, the simulated efficiency of the 1 kW synchronous H6 is up to 2% higher at 20% load. This gap between simulated and experimental results may be attributable to accuracy of the measurement equipment, as controller and snubber circuitry losses were taken into account in the simulation. It is important to also note that the power loss caused by the utilized snubber circuit in the bidirectional synchronous H6 inverter is proportionally increased when the operating frequency is increased, particularly when the inverter is subjected to light-load conductions.

## 5. Conclusion

In this paper, the optimal design of a 5 kW bidirectional synchronous H6 inverter suitable for hybrid AC/DC distribution systems for residential buildings is simulated and analyzed, and the effects of SiC and Si power MOSFETs operating at different frequencies are compared. By using SiC instead of Si, the efficiency of the synchronous H6 inverter can be improved, particularly at light-load. A SiC topology provides a predicted efficiency of up to 98.3% versus 93.6% for loads under 20% for a 5 kW system when compared to Si. Similarly, the predicted and measured efficiency of a 1 kW system demonstrates the same trend under light-load conditions. The improvement is a result of reduced SiC conduction and inductor loss under various load conditions.

However, while the size of the inductor is decreased, the overall efficiency provided by the increased frequency decreases at light-load and improves slightly at full load. Given the trade-off between high switching loss and lower copper loss as frequency increases, the optimal operating frequency for the synchronous H6 is 20 kHz. The results of the experiments at light-load conditions (20% of its maximum power) validate the level of improvement in the efficiency of the 1 kW system and confirm that a similar level of improvement can be achieved with the 5 kW system.

Findings highlight the significance of the reverse recovery effect in the synchronous H6 for Si and show how SiC may be used to mitigate this issue. However, it should be noted that the cost of SiC is approximately triple that of Si for the same voltage and current ratings with an estimated value of 3.6 euros, in comparison to 0.9 euros for specific SiC and Si MOSFETS, so the reduction in losses provided by the higher efficiency should be sufficient to compensate for this higher cost. The high switching loss of the Si solution is being worked on, with diode emulation being considered so that it can perform as a standard H6.
